# Evaluation and screening of peanut (*Arachis hypogaea* L.) germplasm for nutritional and biochemical enhancement with in-vitro assessment of anti-cancer properties

**DOI:** 10.1371/journal.pone.0339289

**Published:** 2025-12-30

**Authors:** Sania Kabir, Mahmood ul Hassan, Muhammad Kausar Nawaz Shah, Kashif Sarfraz Abbasi, Ata-ur Rehman, Kenneth Chinkwo, Muhammad Zargham Ali

**Affiliations:** 1 Department of Plant Breeding and Genetics, PMAS- Arid Agriculture University, Rawalpindi, Pakistan; 2 School of Dentistry and Medical Sciences, Charles Sturt University, Wagga Wagga, New South Wales, Australia; 3 Institute of Food & and Nutritional Sciences, PMAS- Arid Agriculture University, Rawalpindi, Pakistan; 4 Department of Agrobiotechnology, Justus Liebig University, Gießen, Germany; University of Balochistan, PAKISTAN

## Abstract

Peanut breeding increasingly emphasizes nutritional enhancement and bioactive potential to support functional food development. This study assessed twenty-eight peanut genotypes for compositional diversity and invitro anticancer and attributes.Principal component analysis revealed 47.78% of the total variation among genotypes, primarily influenced by protein,oleic acid and phenolic content. Significant variation was observed in fatty acid and phenolic profiles revealing genotypes with superior nutritional and antioxidant properties. High phenolics and antioxidant rich genotypes were further analyzed using invitro assay (Resazurin-red) suggesting possible anticancer effects. LC/MS profiling identified 11 phenolic compounds such as p-coumaric acid and quercetin, associated with antioxidant and chemopreventive effects. Extract from the most bioactive genotypes effectively reduced colon cancer cell viability, suggesting possible anticancer potential. All the genotypes have maintained aflatoxin levels below safety thresholds, confirming their suitability for food use. Overall the findings highlight the potential of nutritionally enhanced peanut germplasm for developing dual purpose varieties with improved health and dietary benefits. These results provide a valuable foundation for breeding programs targeting functional and chemopreventive peanut cultivars. However, further in vivo validation of phenolic-driven anticancer mechanisms is warranted to advance functional peanut varieties.

## 1. Introduction

Oil seed crops comprise a vital segment of Pakistan’s agriculture [1]. Peanut (*Arachis hypogaea* L, groundnut) belongs to the family Fabaceae and is an important oilseed and food legume crop valued for its edible oil and protein content. It is grown in over 100 countries, producing approximately 47 million tons annually from around on 20 million hectares of land [2]. The crop is believed to have originated in Central and South America, with cultivation spread to Asia and Africa which now account for nearly 70% of global production [3]. China and India remain the leading producers followed by the United States and Argentina [4]. In Pakistan, 84% of the peanut cultivated area lies under the Punjab province, particularly across the rainfed Pothohar plateau encompassing Attock, Chakwal, Jehlum, and Rawalpindi districts [5].

In addition to being an important oil crop, peanuts are widely consumed due to their flavor, affordability and nutritional richness [6].Peanut seed contains 40–56% of edible oil, 20–30% protein, and 10–20% carbohydrate making them highly nutritious food source [7].Kernels are a good source of vitamin E and other micronutrients such as niacin, calcium, magnesium, phosphorus, zinc and potassium [8]. Among these, vitamin E and its derivatives possess strong antioxidant potential and has been proven to modulate signaling molecules that regulate oxidative balance [9].

They also contain a wide range of bioactive phytochemicals with health-promoting properties, including sterols, tocopherols, unsaturated fatty acids, and phenolic compound. Phenolic compounds such as hydroxybenzoic acid, ferulic acid, coumaric acid, resveratrol, flavonoids (catechin and procyanidins), and flavanols (quercetin and kaempferol) have been identified in peanuts kernels [10]. New findings suggest that a huge number of flavonoids are strong antioxidants that help to balance the reactive oxidative species thus minimize oxidative stress [11]. These compounds, along with condensed tannins and other polyphenolics, exhibit antioxidant exert anti-inflammatory, anti-adiposity, and anti-cancer activities [12].A recent study declares the boiled peanut as a significant source of resveratrol in comparison to other roasted or raw parts of peanut [13]. Resvratrol (a stilbene compound) is known for its antioxidant and chemoprotective properties [14]. Polyphenol antioxidants have now been linked to minimize heart diseases, inflammation, cancerous tumors and neurological complications [15].

Modern day ailments are believed to result from prolonged oxidative stress in the human body. In present, cancer is one of the deadliest diseases in the world and in most developed countries, colon cancer is one of the most common ones closely associated with food intake choices and everyday lifestyle practices [16].Traditional cancer treatments such as chemo and radiotherapy are effective at destroying malignant cells but they often cause severe effects due to their non-selective cytotoxicity (damaging healthy tissues along with cancer cells). These treatments can also induce oxidative stress, inflammation and immune suppression which can hinder recovery and reduce patient’s quality of life [17]. In contrast, phenolic compounds naturally present in peanuts act through multiple molecular pathways that selectively target cancer cell mechanisms without harming normal cells [18].

Although peanuts are widely recognized for their nutritional worth, little is known about their phenolic composition and potential anticancer properties. The scarcity of research, particularly against colon cancer underscores the need to evaluate peanut germplasm for phenolic diversity and functional helath promoting varieties. Therefore, this study aims to evaluate and characterize diverse peanut germplasm for their nutritional and biochemical composition, and to access their in vitro anticancer potential against human colon cancer cells to identify promising genotypes for functional food and crop improvement. Peanut phenolic compounds were examined in this research as they are usually considered as more important but neglected part of the bioactive compounds. The most promising genotypes based on highest antioxidant activity were then tested for their power to inhibit the overall cell viability of HT-29 human colon cancer cells. Considering the peanut’s biological worth, this study contributes in establishing a primary pathway to assess its in vitro potential for anticancer properties. These findings can strongly support the participation of bioactive compounds in peanut extracts in minimizing the development and progression of cancer.

## 2. Materials and methods

### 2.1 Germplasm collection

Twenty-eight genotypes of peanut (*Arachis hypogaea* L.) were obtained from United States Department of America (USDA-ARS mini core collection), Plant Genetic Resources Conservation Unit (PGRCU) Griffin, USA), Pakistan and China shown in Table 1.

**Table 1:** Peanut germplasm acquired from USA, China and Pakistan used for this study.

Genotype Code	Original ID	Source
V1	PI 619175 01 SD	USDA
V2	PI 594923 01 SD	USDA
V3	PI 564845 01 SD	USDA
V4	PI 639691 01 SD	USDA
V5	PI 658102 02 SD	USDA
V6	PI 635006 01 SD	USDA
V7	PI 565434 01 SD	USDA
V8	PI 481776 01 SD	USDA
V9	PI 478784 01 SD	USDA
V10	PI 383431 03 SD	USDA
V11	PI 383426 02 SD	USDA
V12	PI 383424 01 SD	USDA
V13	PI 267772 01 SD	USDA
V14	PI 478787 01 SD	USDA
V15	PI 564846 01 SD	USDA
V16	PI 542961 01 SD	USDA
V17	PI 564844 01 SD	USDA
V18	PI 544346 02 SD	USDA
V19	PI 564847 01 SD	USDA
V20	BARI 2000	BARI, Pakistan
V21	Golden	BARI, Pakistan
V22	BARI-2011	BARI, Pakistan
V23	Chinese Black	China
V24	Chakori	BARI, Pakistan
V25	Potohar	BARI, NARC
V26	Chinese (1)	China
V27	Chinese (2)	China
V28	Chinese (3)	China

Peanut samples obtained from the United States Department of America (USDA-ARS mini core collection), Plant Genetic Resources Conservation Unit (PGRCU) Griffin, USA), Pakistan and China.

### 2.2 Extraction and evaluation of the oil

Peanut oil was extracted from 10 g of ground peanuts for each genotype using n-hexane as a solvent in a Foss Soxtec 2050 apparatus according to the method of the ISO 659 ‘Oilseeds – Determination of oil content based on AOCS official method Ai 3–75, involving a single grind and 16 hours extraction at 120°C ± 10°C [19].

### 2.3 Estimation of protein content

Crude protein content in the peanut samples was determined using the LECO TruMac CN apparatus, following the AOCS official method (99.23). Nitrogen levels were quantified and multiplied by a factor of 6.25 to estimate protein content [20].

### 2.4 Evaluation of fatty acid profile

The fatty acid profiles of 28 genotypes was analyzed using an Agilent Gas Chromatograph for their according to the AOCS method (Ce 1–62) [21]. The fatty acids in peanut samples were mentholated into fatty acid methyl esters (FAMEs). The % Area of selected fatty acids from the chem station offline software was estimated.

### 2.5 Estimation of aflatoxin content using ELISA

Aflatoxin content in peanut samples was quantified using an Enzyme-Linked Immunosorbent Assay (ELISA) following the method adopted by Waliyar et al. [22]. The ELISA kit (2–50ppb range) was sourced from Romer Labs Singapore Pte. Ltd. All raw peanut samples were inoculated with *Aspergillus flavus.* The A. flavus strain was procured from Barani Agriculture Research Institute (BARI), Chakwal, Pakistan, following the protocol described by Nataranjan et al. [23].

### 2.6 Evaluation of antioxidant potential

#### 2.6.1 Sample preparation for evaluation of phenolic content and antioxidant activity.

Phenolic compounds were extracted following a previously standardized method conducted by the same group of authors and same working conditions [24]. The extraction solvent was prepared with acetone, water and acitic acid solution in ratio (70:29.5:0.5v/v/v) was added at ratio of 1:10 (w/v) to the peanut defatted flour. After 1 hour of stirring at room temperature, the mixture was centrifuged at 4000 rpm for 10 minutes. Sample was pooled to collect the supernatant by repeating the step. Acetone was evaporated from the collected sample using rotavapor (R-210 BUCHI Labortechnik, Flawil, Switzerland). Sample was kept at −80°C to remove the remaining liquid and then kept at −20°C after freeze drying until further analysis. For phenolic compounds analysis, the dried peanut sample was reconstituted in 80% methanol. However, for cell culture experiments, the phenolics crude dried extract was reconstituted in 50% DMSO.

#### 2.6.2 Estimation of Total Phenolic Contents (TPC).

The TPC was quantified using the Folin-Ciocalteu method, as reported by Salve et al. [25]. The absorbance was read at 750 nm using a 96-well microplate with the Fluo star Omega BMG multi-detection microplate reader. The results were expressed as mg Gallic acid equivalent/ gram of dry sample.

#### 2.6.3 DPPH radical scavenging capacity.

The potential to scavenge DPPH was estimated using method followed by Zhou et al. [26]. The absorbance was measured at 517 nm. Trolox as a standard was used for calculations (18.5 to 300 mg/g), y = −0.0228x + 0.9655, R2 = 0.9999). Control (without extract) was also made in the same way, and methanol was used for baseline correction. DPPH Radical scavenging of peanut genotypes was stated as mg Trolox equivalent per gram of dry peanut sample.

#### 2.6.4 Ferric Reducing Antioxidant Power (FRAP) assay.

FRAP assay was conducted according to the method followed by Zhou et al. [26]. The results were expressed in mg of Trolox equivalent antioxidant capacity per gram of dry sample.

#### 2.6.5 Determination of individual phenolic acids using LC/MS-QTOF.

Individual phenolic compounds in peanuts were identified using Agilent Technologies 6530 Accurate- Mass Q-TOF LC/MS.. Peanut dried phenolic extracts were dissolved in 80% methanol (1 g/ml). The compounds were separated at room temperature using a C18 110A column (150 x 4.6 mm). The mobile phase comprised 0.1% formic acid (F.A.) in deionized water as solvent A and 80% methanol as solvent B. The mobile phase flow rate was fixed at 1.0 ml/min, and a sample volume of 20 µL was injected. The detection of phenolic compounds was observed at 280 nm. An online ABTS system was run to assess the antioxidant potential of the identified compounds.

### 2.7 Evaluation of antiproliferative activity of peanut phenolic extracts

#### 2.7.1 Cell culture.

Extraction solvents used in cytotoxicity assay including acetone and acetic acid were sourced from Chem Supply Pty Ltd. (South Australia, Australia). Dulbecco’s modified eagle’s medium (DMEM), fetal bovine serum (FBS), Dimethyl sulphoxide (DMSO), penicillin-streptomycin, trypsin, phosphate buffer saline (PBS), resazurin, hydrogen peroxide (H_2_O_2_) and stain buffer were obtained from Sigma-Aldrich (St. Louis, MI, USA).HT-29 cells were initially purchased from sigma- Aldrich (St Louis, Missouri, USA) and were previously well maintained by similar group of authors in the same laboratory conditions. Cells were further grown and maintained using DMEM and supplemented with 10% FBS and 1% penicillin-streptomycin. All cells were grown in a humidified 5% (v/v) CO2 incubator at 37°C. Cell line HT-29 with passage number 2–7 was used in this study.

### 2.8 Resazurin red cytotoxicity assay

For in vitro cytotoxicity assessment of phenolic peanut extracts, a time/dosage dependent assay was conducted. The samples were dissolved in dimethyl sulphoxide (DMSO) and diluted further to get concentrations ranging from 500–2000µg/ml followed by Bochenek et al. [27]. Human colon cancer cells (HT-29) were cultured in 96-well plates with Dulbecco’s Modified Eagle Medium (DMEM) medium. In contrast, the cell density was maintained at 5 × 104 per well and left overnight in incubation to achieve the desired confluence (85–90%). A (mechanistic) positive control of 10mM Hydrogen peroxide (H_2_O_2_) was used. In contrast,0.5% DMSO (the maximum level present in reconstituted extracts) was used as a (vehicle) negative control, and no cells were used as blank. After 24 hours, the medium was discarded carefully, leaving the cells adhering to the bottom of each well. Phosphate-buffered saline (PBS) was used to wash the cells. The cells were then exposed to different concentrations of peanut extracts and incubated again at 37 °C. To quantify the number of live epithelial cells in the sample, 100 μL of resazurin dye was added in darkness and incubated for 4 hours. The cells were counted at intervals of 12 hours and 24 hours. Resazurin red dye was prepared following the method of Franchis et al. [28]. The absorbance values were recorded at 570 and 600 nm using the microplate spectrophotometer reader Multiskan GO (Thermo Fisher Scientific). The data show the mean ± standard deviation (S.D.) of three wells per treatment and represent three experiments.

### 2.9 Statistical analysis

The data were statistically computed by means of a one way and two way analysis of variance (ANOVA) using R-studio (4.3.2) according to Steel and Torrie [29]. The results are expreassed as means ± standard deviation. Least Significant Difference (LSD) according to Federer, Nair (55) at a 5% significance level to assess the differences among genotypes and facilitate the identification of specific genotype pairs exhibiting significant differences. In addition, Principal Component Analysis (PCA) was employed using the “FactoMineR” package of R-studio to elucidate the major factors contributing to variation within the genotypes. A dendrogram was constructed to depict the hierarchical clustering of genotypes, providing a visual representation of genetic relationships and similarities among them. Biplot and heatmap analysis was also performed for a visual representation of the diversity and correlations among traits. Resazurin red assay was appropriately applied to the used concentrations of phenolic compounds after the treatment of compounds to the controls. The graphs were created using the ggplot2 package of R studio for antioxidant activity and GraphPad Prism 7.0 software for comparison of viability percentage of cancer cells.

## 3. Results

### 3.1 Protein content

**Fig 1:**
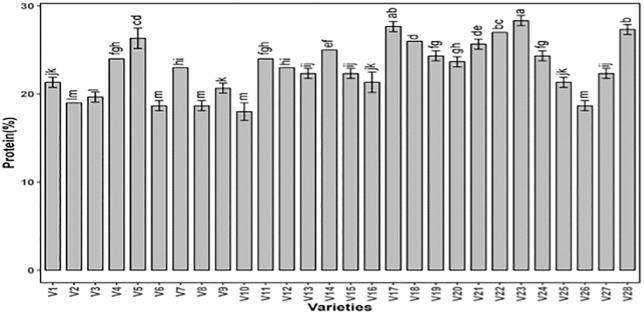
Estimation of total protein content in twenty-eight peanut genotypes. The data presented are the mean ± SE; different alphabetic letters represent significant differences at 5%.

The analysis of variance for protein content revealed substantial significance among genotypes (Table 1). The overall protein content in tested peanut genotypes was noted high. However, the genotype V23 appears to have the highest protein content (28.3%). In contrast, genotypes such as V6, and V10 show relatively lower protein contents (17.67%).The higher protein content indicates that these promising genotypes can be exploited in breeding programs aimed at developing high peanut cultivars to enhance nutritional value, primarily in the regions where peanut serves as a source of dietary proteins [30] (Fig 1).

### 3.2 Fatty acid profile

**Fig 2:**
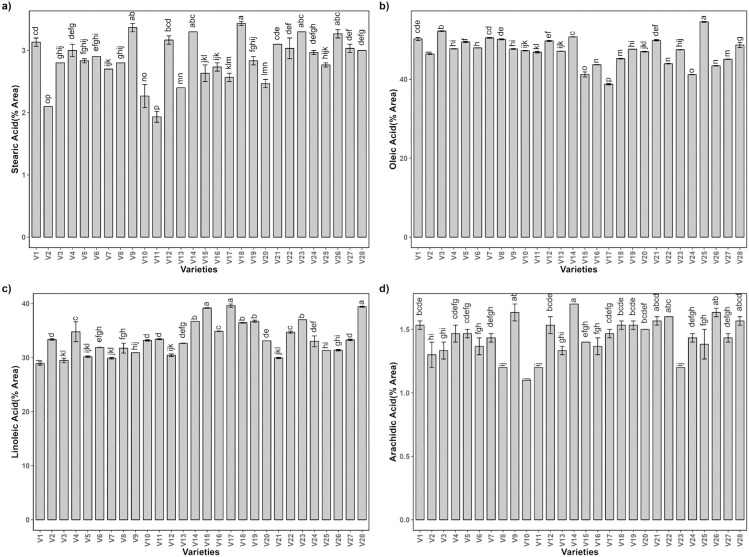
Fatty acid profile of twenty-eight peanut genotypes using Agilent Gas Chromatograph, a) Stearic acid b) oleic acid c) Linoleic acid and d) Arachidic acid. The data presented are the mean ± SE; different alphabetic letters represent significant differences at 5%.

A diverse array of fatty acids was quantified, with oleic, linoleic, palmitic, stearic, myristic, and arachidic acids being the most prevalent. Significant variations were observed, in oleic, linoleic, stearic, and arachidic acid shown in (Fig 2), as evidenced by ANOVA, (Table 2). The content of stearic acid varies among the genotypes, with concentrations ranging from approximately 1.5 to 3.5 percent. Genotype V18 exhibits the highest stearic acid content, whereas V2 and V11 show relatively lower levels. The oleic acid levels appear more uniform across genotypes than stearic acid, with most genotypes exhibiting values around 40 percent. A few genotypes, such as V2 and V25, have slightly higher oleic acid content. Higher oleic acid content is desirable for both human health and shelf life of peanut oil and its derived products, due to association with improved lipid metabolism and reduced cardiovascular risk respectively and for industry, as it enhances oxidative stability [31]. Linoleic acid content has a notable variation, with values spanning from about 25 to almost 40 percent. Genotypes V15, V17, and V28 are particularly high in linoleic acid, indicating a potential genetic predisposition for this fatty acid accumulation. The arachidic acid concentration across the genotypes shows moderate variation ranging from roughly 1.0 to 1.5 percent. Some genotypes, such as V10 and V23, have lower levels, whereas V9 and V14 are among the highest.

**Table 2:** Analysis of variance among twenty-eight peanut genotypes for selected nutritional quality parameters including protein content, fatty acids profile, DPPH, FRAP, TPC and aflatoxin content.

Traits	Mean (SE)	LSD	Minimum	Maximum
Protein (%)	27.3 ± 0.33***	0.94	18	28.3
Stearic Acid (%)	2.85 ± 0.07 ***	0.2	1.93	3.4
Oleic Acid (%)	47.21 ± 0.21***	0.6	38.76	54.56
Linoleic Acid (%)	33.47 ± 0.45***	1.29	28.9	39.6
Arachidic acid (%)	1.43 ± 0.04***	0.13	1.1	1.7
DPPH (mg/g, Trolox eq.)	5.44 ± 0.04***	0.13	5.01	5.91
FRAP (mg/g Trolox eq.)	8.19 ± 0.37***	1.05	4.04	17.7
TPC (mg/g, G.A eq.)	111.38 ± 1.04***	2.96	57.64	159.27
Aflatoxin (ppb)	3.04 ± 0.05***	0.13	0.07	11.71

The data presented are the mean ± SE (n = 3). Significant difference between traits is represented by * and significance was tested at 5%.

### 3.3 Aflatoxin content

**Fig 3:**
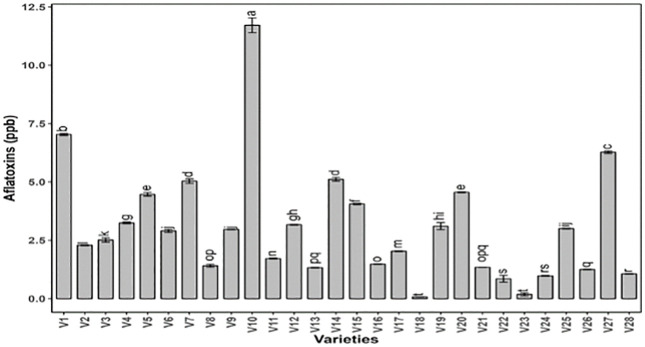
Comparison of mean values for aflatoxin content present in twenty-eight peanut genotypes. The data presented are the mean ± SE; different alphabetic letters represent significant differences at 5%.

All genotypes exhibited highly significant variation for aflatoxin content, and the results for ANOVA are presented in (Table 1). The genotype V18 was marked as the best with minimum aflatoxin content (0.0667 ppb), whereas V10 possess the highest (11.717 ppb), aflatoxin content, (Fig 3).This particular trait can be helpful to evaluate the differences in susceptibility and resistance to fungal infections and production of different toxins [32].

### 3.4 Principal component analysis and heat map analysis

**Fig 4:**
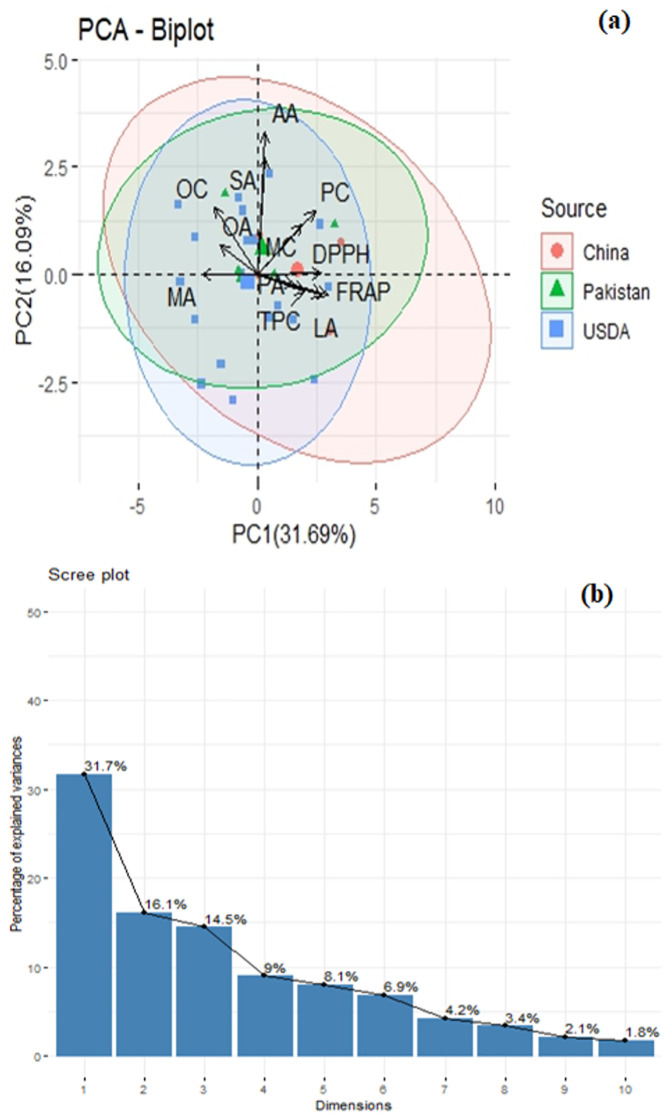
Principal component analysis (PCA) of twenty-eight peanut genotypes (A) Biplot analysis of genotypes gathered from distinct origins based on their quality and nutritional parameters, (B) Scree plot.

The principal component analysis was performed based on first two quardrants. The PCA biplot, (Fig 4A) elucidates the relationships within the data, where the proximity of parameter vectors indicates potential correlations. Four traits such as oleic acid (OA), stearic acid (SA), oil content (OC) and protein content (PC) were grouped together indicating their positive relativity, genotypes high in any one of these characteristics tend to be high in the others. Breeders can choose these traits together to improve both oil quality and nutritional worth at the same time. Linoleic acid (LA) and total phenolic content (TPC) were located separately, suggesting that they vary independently from other group and can be used focussed in targeted breeding as a single trait improvement.

**Fig 5:**
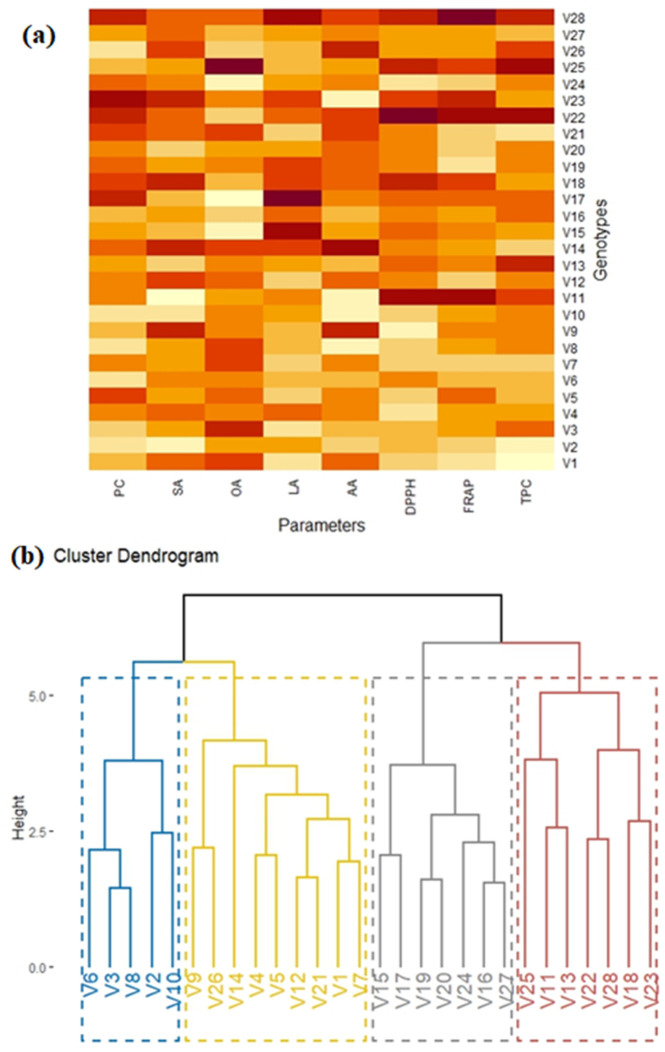
Biochemical diversity among a panel of 28 peanut genotypes: (A) Heat map showing intensity and variation among genotypes for selected traits, (B) Hierarchical clusters dividing peanut genotypes into two major and four minor groups based on similarity among genotypes.

The second principal component contributes an additional 16.1% to the explained variance, cumulatively accounting for nearly half of the total variance using only two components as shown in (Fig 4B). Heat map and dendogram divides the genotypes into four main clusters based on biochemical similarities as shown in (Fig 5A). This selection of traits was used for detailed analysis due to their substantial contribution to the overall variability. Additionally, the biplot reveals clustering tendencies among genotypes from different origins.

The hierarchical cluster dendrogram categorizes the twenty-eight peanut genotypes into four major clusters, as denoted by distinct color-coded branches as shown in (Fig 5B). These clusters represent groupings based on the similarity of their biochemical profiles. The blue cluster (genotypes with similar oil and protein traits) comprises genotypes V3, V6, and V8, indicating a potential closeness in their biochemical attributes. The red cluster, containing genotypes V18, V22, V23, and V28, distinguishes itself as a separate, distinct group, indicating unique biochemical profiles helpful for introducing new variability. The grey and yellow clusters exhibit a higher degree of diversity, with their subclusters suggesting subtle relationships among their constituent genotypes. These can be good sources of broad-based breeding populations that combine diverse desireable characteristics. The genotypes from China, Pakistan, and the USDA are marked with red squares, green triangles, and blue diamonds, respectively, and are grouped within distinct ellipses. The overlap among these genotypes indicates shared biochemical characteristic, possibly due to common ancestory or similar selection pressure.

### 3.5 Total Phenolics Contents (TPC)

Total phenolic content of peanut genotypes was tested using the Folin-Ciocalteau reagent. The values were found in the range of 103–157.7 mg/g gallic acid (G.A.). Genotype V18 showed a minimum total phenolic content and genotype V25 showed a maximum value, i.e., 157.7 (mg/g). The detailed results for significant differences are shown in (Table 2).

### 3.6 DPPH radical scavenging capacity

Peanut genotypes (V11, V18, V22, V23, V25, and V28) dominated in the initial screening, with the values recorded more than average. Genotype V22 showed maximum (5.94 mg/g) antioxidant activity by DPPH radical scavenging assay, while genotype V23 showed a minimum value (5.00 mg/g). Significant differences among all genotypes are shown in (Table 2).

### 3.7 Ferric Reducing Antioxidant Power (FRAP) assay

Significant results for antioxidant activity tested by FRAP assay for peanut genotypes showed the values recorded more than average (Table 2). Genotype V28 was found to be on the top with a maximum value of (17.87 mg/g), and genotype V18 showed a minimum value of 10.82 mg/g.

### 3.8 Determination of individual phenolic compounds by UHPLC/MS-QTOF

**Fig 6:**
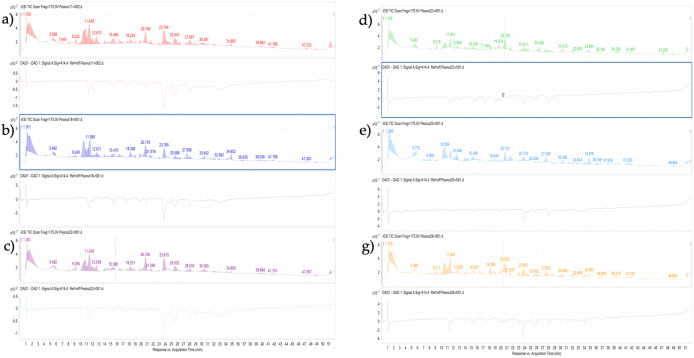
Identification of major phenolic compounds and their relevant chromatographic peaks in methanol extracts of peanut genotypes through Mass Q-TOF LC/MS with online ABTS system. The compounds were separated using the C18 110A column (150 x 4.6 mm): mobile phase comprised 0.1% formic acid (F.A.) in deionized water as solvent A and 80% methanol as solvent B. The mobile phase flow rate was fixed at 1.0 ml/min, and a sample volume of 20 µL was injected. The detection of phenolic compounds was observed at 280 nm. An online 2,2′-azino-bis (3-ethylbenzothiazoline-6-sulfonic acid) (ABTS.+) system was run to assess the antioxidant potential of the identified compounds: (A) Genotype V11; (B) Genotype V18; (C): Genotype V22; (D) Genotype V23; (E) Geno type V25; (F) Genotype V28.

A total of eleven phenolic compounds were identified from peanut extracts when subjected to LC/MS analysis, as shown in (Table 3). Peak identification of phenolic acid in peanut extracts is shown in (Fig 6A–6C)which includes genotypes V11, V18 and V22 while (Fig 6D–6F) icludes genotypes V23, V25 and V28 respectively (Table 4).

**Table 3:** Total phenolic content and antioxidant activity of six selected peanut genotypes based on maximum values shown by TPC, DPPH, and FRAP assays.

Selected Genotype	TPC (mg/g, GA eq.)	DPPH (mg/g, Trolox eq.)	FRAP (mg/g, Trolox eq.)
V11	132.7 ± 1.95 ^def^	5.85 ± 0.04 ^ab^	15.63 ± 0.33 ^b^
V18	103.7 ± 3.45^j-n^	5.83 ± 0.01 ^ab^	10.82 ± 0.26^e^
V22	153.7 ± 9.85^a^	5.94 ± 0.11 ^a^	13.68 ± 0.25^c^
V23	110.5 ± 12.68^h-m^	5.73 ± 0.01 ^bc^	12.48 ± 0.15^d^
V25	157.7 ± 11.07^ab^	5.79 ± 0.01^ab^	12.20 ± 0.20^d^
V28	148.9 ± 14.61^abc^	5.75 ± 0.01^b^	17.87 ± 1.04^a^

The data represent each sample’s mean ± SD of the triplicate assay. The significance of each peanut cultivar was compared at *P* < 0.05. The absorbance was read at 750 nm and 517 nm for total phenolic content and antioxidant activity respectively.

**Table 4:** Peak areas of key bioactive phenolic compounds characterised in peanut genotypes. The values are presented after log transformation for Analysis of Variance, and the P values are for the multiple comparisons between varieties. ND represents where compounds were not detected in the sample.

Parameters Varieties	PBA	CuA	FeA	CGA	CAT	QC	KP	LU	CA	SA	DA
V11	3.2 ± 1.4^c^	3.3 ± 2.6^d^	N.D.	N.D.	2.9 ± 3.5^e^	2.5 ± 3.4^d^	2.5 ± 0.1^d^	N.D.	2.8 ± 0.9^d^	6.2 ± 0.6^b^	2.7 ± 1.7^c^
V18	6.0 ± 0.1^b^	5.8 ± 0.2^c^	N.D.	N.D.	6.3 ± 1.1^c^	5.2 ± 2.9^b^	2.8 ± 3.9^c^	2.8 ± 0.3^c^	5.1 ± 0.3^c^	6.2 ± 0.3^b^	4.9 ± 0.1^b^
V22	3.2 ± 2.5^c^	2.9 ± 2.0^e^	N.D.	N.D.	3.6 ± 3.6^d^	2.6 ± 1.3^d^	2.4 ± 3.3^d^	2.4 ± 1.5^d^	N.D.	3.2 ± 1.4^d^	2.5 ± 1.5^d^
V23	5.8 ± 0.3^b^	6.1 ± 0.1^b^	3.3 ± 0.3	2.4 ± 0.2	6.7 ± 0.2^b^	5.8 ± 0.2^a^	5.1 ± 0.2^a^	5.1 ± 1.1^a^	5.4 ± 0.1^b^	5.8 ± 0.3^c^	N.D.
V25	6.5 ± 0.1^a^	6.6 ± 0.2^a^	N.D.	N.D.	2.8 ± 0.1^e^	4.5 ± 0.1^c^	4.5 ± 0.1^b^	4.5 ± 3.6^b^	2.6 ± 1.6^e^	6.5 ± 0.4^a^	2.7 ± 1.7^c^
V28	6.6 ± 0.2^a^	5.9 ± 0.1^c^	N.D.	5.6 ± 0.1	7.1 ± 0.1^a^	5.9 ± 3.7^a^	2.6 ± 1.7^d^	2.6 ± 0.1^d^	5.5 ± 0.1^a^	6.6 ± 0.2^a^	5.0 ± 1.5^a^
P-value	4.4	3.9	---	3.4	4.1	3.8	2.7	1.01	2.5	3.3	2.4

**Key:** PBA: P-hydroxybenzoic acid, CuA: Coumaric acid, FeA: Ferulic acid, CGA: Chlorogenic acid, CAT: Catechin, Quercetin, KP: Kaempferol, LU: luteolin, CA: Caffeic acid, SA Salicylic acid DA: Daidzein and ND: Not Detected.

### 3.9 Antiproliferative activity of peanut phenolic extracts

**Fig 7:**
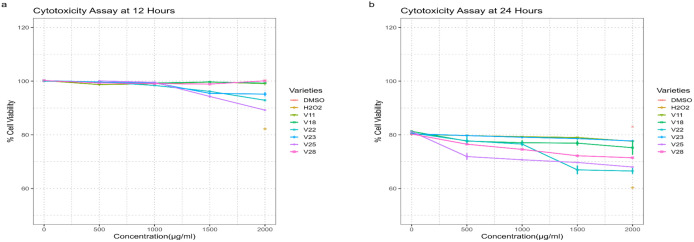
Cytotoxicity effects of six peanut phenolic extracts on human colon cancer cell line HT-29 tested by resazurin red cytotoxicity assay using different concentrations (500, 100, 1500 & 2000) µg/ml. DMSO was used as a negative control, and H_2_O_2_ was used as a positive control. Results represent mean ± standard deviation; (A) Effect of extracts on HT-29 colon cancer cell viability (%) at 12 hours; (B) Effect of extracts on HT-29 colon cancer cell viability (%) at 24 hours.

A time dose-dependent cytotoxicity assay using resazurin red dye was established to investigate the impact of peanut phenolic extracts on HT-29 cells. Human colon cancer cells HT-29 were treated with phenolic extracts of different peanut genotypes at concentrations of 500, 1000, 1500, and 2000 µg/ml, compared at 12 and 24-hour intervals. Results for both time points (12 hours and 24 hours) are presented in (Fig 7A and 7B), respectively. Hydrogen peroxide (H_2_O_2_) 10M, and 0.5% DMSO were used as positive and negative controls, respectively, and extracts with no cells were used as a blank. The primary focus of this study was based on the mechanism-oriented screening rather than direct preclinical potency comparison, established chemotherapeutic agents were not used as benchmark anticancer drugs. Inclusion of such standards will be addressed in follow-up studies to quantify comparative potency.

Genotypes V22 and V25 were recorded as outstanding among all tested extracts. Fig 7A demonstrates the significant reduction in cancer cell viability percentage in genotype V22 at 12 hours at higher dosages such as 1500 and 2000. However, at 24 hours, (Fig 7B) the same extract (genotype V23) showed a noticeable response at all tested dosages (500−2000 µg/ml). Similarly, genotype V25 showed a significant drop in percentage cell viability at the lowest concentration when observed after 24 hours compared to 12 hours. Extracts of two peanut genotypes, V11 and V18, exhibited the least potency against HT-29 cancer cells without significantly decreasing cancer cell viability at any tested dosage on both time points.

## 4. Discussion

Highly significant results were obtained for fatty acid profile, protein content, TPC, DPPH, FRAP and aflatoxin content contributing to the maximum overall diversity among 28 peanut genotypes. Six selected genotypes were then subjected to their inhibitory effect against HT-29 human colon cancer. The diversity in fatty acid profiles, particularly in stearic, oleic, linoleic, and arachidic acids, is a pivotal finding, considering their nutritional relevance. This diversity echoes previous research by Gulten et al. [33], highlighting the crucial roles these fatty acids play in human health. Oleic acid is an essential parameter to assess food superiority and wellbeing in the context of human health. Another study, emphasizing the predominance of oleic and linoleic acids in these genotypes, adds to the evidence supporting their nutritional value, aligning with recent dietary trends that favor these fatty acids for their health benefits [34]. High oleic acid containing genotypes found in this study can be given a priority in recent peanut breeding selection programs. Similar results were obtained by Li W et al. [35]. As the development of a high-oleic-content peanut genotype having a diverse genetic background is a matter of importance and will advantage the breeding of peanut for high oleic acid content [36].Also the high oleic acid genotypes can be desirable in breeding programs aimed to produce nutritionally superior and storage stable varieties [37].

Protein levels across the genotypes studied in this research are reinforcing the findings of Sicherer and Sampson [38] and resonating with Goldstein and Reife [39] on the global demand for plant-based proteins. This range was also observed by Wang et al. [40]. Improved protein content also contributes to better amino acid balance and higher nutritional quality, supporting the selected genotypes in food fortification and value-added product development [41]. Interestingly, some high-protein genotypes also exhibited elevated phenolic and antioxidant levels [42], suggesting possible co-selection opportunities for both nutritional and functional traits.

The variation in aflatoxin content across genotypes, was observed within the safety limits (50 ppb) [43]. This highlights the importance of continuous monitoring and quality control in peanut production. A major barrier to peanut import is the quality deterioration, mainly caused by toxins accumulation [44]. Hence, screening low content of aflatoxin in peanut genotypes can be helpful for maintaining food safety and meeting world regulatory standards [32]. The lower aflatoxin levels observed may be attributed to the low moisture content and gamma irradiation during the quarantine process, as suggested by other studies [45,46]. Our results are also in accordance with the results concluded by other studies according to which the low moisture content leads to the lower contamination level of aflatoxin in maize [47]. Genotypes representing minimum levels of aflatoxin accumulation, can act as a worthy parental source in peanut breeding programs highlighting the purpose of increased *Aspergillus* resistance [48].

An interesting fact about seed coat colors of peanut is that lighter colors like beige represent more antioxidant activity while darker skin colors like pink and purple indicate higher content of anthocyanins as described by Machado et al. [49].Another finding supports the fact that peanut testae colour is an essential factor in determining total phenolic content in which the pink, grey, and yellow colour of peanut testae, concluding that the darker colour (pink) had the maximum TPC compared to yellow and grey [50].

This study also observed similar kind of pattern as genotypes V22, V23, V25, and V28 were darker in colour (dark pink and black–dark purple) and V25 presented maximin value, while genotypes V11 and V18 were lighter pink to white hence V18 was recorded as minimum. This phenomenon was also supported by Kaopha et al. [51], who studied TPC in peas and different legumes, including lab beans, cowpeas, chickpeas, and common beans. He concluded that a positive correlation exists between lighter colour beans and pea varieties with low TPC values and vice versa. The higher TPC values of peanut genotypes indicate the presence of diverse phenolic compounds [52]. This trait worth practical breeding value as a prominent marker for selection of nutritionally rich and potentially stress tolerant peanut lines [53]. According to a recent finding the breeding strategies aiming improvement in nutritional potential and stress tolerance should priorities considering biochemical analysis as they have noticeable genetic advancement along with high heritability and stability across environments [54].

This study is the first demonstration of the effectiveness of peanut phenolic extracts as a functional ingredient to reduce the cytotoxicity of HT29 human colon cancer cells. Plants possess diverse classifications of phenolic compounds, including phenolic acids, simple phenolics, anthocyanins, and flavonoids. These phenolic groups are excellent proton and electron donors due to their unique chemical structure [55].They have achieved excessive attention due to their characteristic physiological functions, such as the capacity to scavenge free radicals, anti-cancerous, anti-mutagenic effects, and anti-inflammatory potentials against different chronic diseases [56].

In this study, with slight modification to reference method, the defatted peanut flour was subjected to the phenolic extraction using 70% acetone which has previously been reported as the strongest and the most effective solvent for phenolic extraction. Furthermore, this makes Folin-Ciocalteu (FCR) method the most accurate method for estimating TPC in peanut phenolic extracts in this case [57]. The TPC values found in this study are comparable to the values recorded by other studies where similar methods were used for phenolic extraction [58]. Another study has presented results similar to our findings while examining Valencia, Spanish, and Virginia-type peanuts [59].

Many physiological processes in the human body driven by the activity of free radicals have been associated with different ailments such as arthritis, cancer, and other inflammatory conditions. To overcome this issue, there is an urge to target more substances with more free radical scavenging capacity and antioxidant potential to significantly inhibit or delay the oxidation process and consequently play a part in disease prevention [60]. Peanut seeds or their by-products have been verified and reported as a generous source of antioxidants [61].Different disease cells, such as cancer cells, are always at an elevated risk of increased and continuous oxidative stress. Therefore, this study suggests that if peanut seeds and other by-products used for different snacks for daily human use would possess antioxidant activity, they could also help prevent cancer.

According to previous studies (chlorogenic, gallic p-hydroxybenzoic, ferulic, caffeic, p-coumaric), flavonoids (luteolin epicatechin, daidzein, kaempferol, and quercetin) and stilbene (resveratrol) are the reported known compounds present in oil seeds such as peanuts [62]. Previous studies have demonstrated that as the concentration of phenolics compound increases, so does their DPPH scavenging activity, presenting the level of antioxidant ability of a representative plant product [63].

The genotypes V23, V25, and V28 were at the top of their TPC values among all genotypes, supporting a positive correlation between the antioxidant activity of the extract and its anti-cancer potential, as mentioned in the previous results concluded by different authors [56]. The seed coat colour of peanuts has also indicated the presence of more phenolic compounds and, thus, more active against various types of cancer [16]. Human nut intake has already been associated with a minimal chance of different cancers and heart diseases [57].

The findings of this research can help build up a baseline for future cancer research studies if peanut could be further investigated for their different biochemical pathways such as oxidative stress modulation, cell cycle arrest and apoptotic induction. There is a huge room to investigate other complex mechanisms that can participate in deterring cancer progression as reported by other plant phenolics such as sorghum, where similar kind of research conducted in the same laboratories where this research has been done concluded that phenolic compounds of pigmented rice and sorghum were effective in activating numerous caspases and expressing p53 [51]. However, functional trait breeding can be an important aspect in future peanut breeding, where integration of biochemical screening into conventional breeding pipelines can combine agronomic performance with nutraceutical value.

This study supports the preliminary evidence of the anticancer potential of peanut extracts against colon cancer cells. Although the exact molecular mechanisms were not investigated, the results suggest possible involvement of antioxidant and pro apoptotic pathways. Moreover, further validation is required in future for peanut phenolic extracts on multiple cell lines with lower extract concentrations to assess the nature of multiple apoptotic genes and complex pathways, mitochondrial activity, and cell cycle evaluation during colon cancer to achieve a broader understanding of different molecular pathways underlying the protective health benefits of peanut phenolic compounds.

## 5. Conclusions

This study established a comprehensive baseline on nutritional composition, phenolic diversity and in vitro anticancer potential of diverse peanut germplasm. Selected genotypes (V22, V25, V28), characterized by darker seed coats and high phenolic content (148.9–157.7 mg GAE/g), exhibited dose-dependent cytotoxicity against HT-29 colon cancer cells, underscoring the functional and breeding significance of these bioactive traits. Future research must validate these results in vivo and elucidate molecular mechanisms (e.g., p53/caspase pathways) to advance peanuts as cost-effective dietary adjuvants for cancer prevention. These findings provide a roadmap for future research focused on molecular breeding, validation across multiple cancer cell lines testing lower concentration levels and regressive mechanistic studies. Despite these constraints, the study offers essential preliminary insights to guide the development of nutritionally improved peanut cultivars with potential health promoting benefits.

## Supporting information

S1 File Inclusivity in global research questionnaire (1). (DOCX)

S2 File Supplementary data. (XLSX)
